# Managing sleep problems using non‐prescription medications and the role of community pharmacists: older adults’ perspectives

**DOI:** 10.1111/ijpp.12334

**Published:** 2017-03-06

**Authors:** Olufunmilola Abraham, Loren J. Schleiden, Amanda L. Brothers, Steven M. Albert

**Affiliations:** ^1^ Department of Pharmacy and Therapeutics School of Pharmacy University of Pittsburgh Pittsburgh PA USA; ^2^ Department of Behavioral and Community Health Sciences Graduate School of Public Health University of Pittsburgh Pittsburgh PA USA

**Keywords:** community pharmacists, decision‐making, over‐the‐counter medications, self‐treatment, sleep health

## Abstract

**Objectives:**

To examine older adults’ perspectives regarding managing sleep problems through selection and use of non‐prescription sleep aids, and the role of pharmacists.

**Methods:**

Telephone interviews were conducted from May to June 2015 with 116 individuals aged ≥60 years in Pittsburgh, Pennsylvania. Participants reported in a previous survey to have used at least one non‐prescription sleep aid in the past 30 days and were willing to participate in a follow‐up interview. Interview guides were designed to elicit perspectives of sleep problems, selection and use of non‐prescription sleep aids, and consultation with healthcare professionals. Interview transcripts underwent content analysis.

**Key findings:**

Four themes emerged as follows: experiences with sleep problems, selection of non‐prescription sleep aids, non‐prescription sleep aid use and interactions with healthcare professionals. Over half of participants reported using a non‐prescription sleep aid for >1 year, were satisfied with its use and perceived it improved sleep quality. Participants commonly used an antihistamine‐only sleep aid; 36% of participants self‐recommended their sleep aid; and 16% of participants consulted healthcare professionals. Few participants read medication dosage labels (22%), side effects or warnings (19%), and many reported they disregarded directions. Participants did not typically consult pharmacists about sleep problems (65%) but perceived that they could assist with medication concerns.

**Conclusions:**

Although most participants had favourable perceptions of non‐prescription sleep aids, older adults may be inappropriately using non‐prescription sleep aids to self‐manage sleep problems by frequently disregarding medication labels and directions for safe use. Also, few older adults are discussing their sleep aid selection and use with pharmacists.

## Introduction

Approximately 6.3 million older adults in the United States report having sleep problems,^1^ and nearly one‐third of older adults report <7 hours of sleep per 24‐hour period.^2^ The National Sleep Foundation recommends a healthy sleep duration of seven to nine hours for adults aged 29–64 and seven to eight hours for older adults aged 65 and older.^3^ Commonly reported sleep problems include trouble falling asleep, waking up during the night or too early in the morning and not feeling rested upon waking.^4^ In one study, 26% of recently retired older adults reported experiencing three or more of these sleep problems.^4^ Sleep problems have been associated with multiple negative outcomes among older adults, including falls,^5^, ^6^ cognitive impairment^6^, ^7^ and mortality.^8^, ^9^

Many factors potentially impact proper sleep hygiene including napping during the day, inconsistent bedtime, consumption of stimulants or food too close to bedtime, insufficient exercise and reading or watching TV in bed.^10^ The average older adult has comorbidities which could be detrimental to sleep quality.^11^ Rather than make behavioural or environmental changes, many older adults opt to self‐treat sleep problems using non‐prescription medications which may not address complex sleep‐related physiological and psychological factors.^12^ Non‐prescription sleep aids include over‐the‐counter (OTC) medications and supplements that may be intended to improve sleep and do not require a prescription. In the United States, most non‐prescription medications and supplements can be purchased online or at various retailers, including community pharmacies and grocery stores. A few non‐prescription products have been restricted by the U.S. Food and Drug Administration (FDA) and require pharmacists to supply these products to consumers to ensure they are therapeutically appropriate. However, non‐prescription products containing active ingredients typically found in OTC sleep aids, such as diphenhydramine or doxylamine, are not in this restricted category. Consequently, these sleep aids have fewer access barriers to protect older adults from inappropriate use than those requiring a prescription.^13^

A recent systematic review found a lack of robust clinical evidence for the safety and efficacy of common non‐prescription sleep aids, including first‐line antihistamines such as diphenhydramine or doxylamine and supplements such as melatonin and valerian, to treat occasional disturbed sleep or transient insomnia.^14^ The Beers Criteria for Potentially Inappropriate Medication Use in Older Adults recommends the avoidance of OTC sleep medications containing first‐line antihistamines.^15^ Diphenhydramine and doxylamine have anticholinergic properties that can result in cognitive impairment, hangover effects, dizziness or falls.^16^ Despite these warnings, it was reported in 2013 that 17% of older adults in the United States were currently using antihistamine‐containing OTC sleep medications.^1^ Older adults in the United States are twice as likely as younger adults to use antihistamine‐containing OTC sleep medications for over 20 days.^17^ Melatonin and valerian supplements are also used to treat sleep problems and may have a lower side effect profile than some OTC sleep medications, but evidence for their benefits in treating insomnia has not been confirmed.^18^ Community pharmacists are accessible healthcare professionals that can counsel older adults on effective use of OTC sleep medications and potential risks for drug interactions to improve patient safety.

To our knowledge, little is known about older adults’ perspectives regarding management of sleep problems, their decision‐making process to select and use non‐prescription sleep aids and consultation with healthcare professionals on appropriate selection. Further research could elucidate these decision‐making processes and targeted interventions to improve older adult patient safety by healthcare professionals such as geriatricians and community pharmacists. Through consultation, healthcare professionals may be able to identify specific sleep hygiene factors that could be influencing sleep problems and suggest non‐pharmacological (behavioural and environmental) interventions to improve sleep. Healthcare professionals, such as community pharmacists, can educate older adult patients taking OTC sleep aids on the potential risks and assist them in selecting appropriate products to improve patient safety. It has been recommended that pharmacists have a more significant role in counselling older adults on appropriate use of non‐prescription sleep aids;^19^ however, older adults’ perspectives of pharmacist‐provided counselling on sleep problems and treatment are unknown. Accordingly, the objectives of this study were to examine the perspectives of older adults regarding their sleep problems, their selection and use of non‐prescription sleep aids, and role of healthcare professionals, particularly community pharmacists, in counselling on sleep problems and treatment.

## Methods

### Sample

The study sample was a subset of individuals that participated in a previous mail survey sent in February 2015 to 2064 members of the Pittsburgh Claude D. Pepper Older Americans Independence Center. The Pepper Community Registry includes over 2000 community‐dwelling persons in the Pittsburgh, Pennsylvania region, aged 60 and older who consented to be contacted for participation in Pepper‐approved research studies. The distributed survey was designed to elicit information on older adults’ perspectives of sleep quality and satisfaction, along with methods of alleviating sleep problems such as the use of OTC medications.^20^ From the sample of 1025 participants who returned the completed survey, 148 participants indicated that they had taken at least one non‐prescription sleep aid within the last 30 days and were willing to be contacted to complete a follow‐up interview. Participants using non‐prescription sleep aids both chronically (more than 20 days) and occasionally (<20 days) were included in this sample. This study was approved on 03 October 2014 by the University of Pittsburgh, USA, Institutional Review Board ID#PRO14080412.

### Data collection

Semistructured telephone interviews were utilized in data collection, and participants’ verbal consent was obtained. An interview guide (see Appendix S1) was designed by the research team to elicit older adults’ perspectives on their sleep problems, strategies for improving sleep including the use of non‐prescription sleep aids and consultation by healthcare professionals such as community pharmacists. To ensure validity, the authors piloted and revised the interview questions and three other researchers not involved in data collection provided feedback on multiple iterations to improve clarity. Participants were asked 33 questions and interviewers used probes to clarify questions and gather the most relevant information. Participants were asked at the beginning of the interview to have their non‐prescription sleep aids available to report the name(s) and directions. Trained researchers from the University Center for Social and Urban Research at the University of Pittsburgh conducted the interviews between May and June 2015, each lasting approximately 10–15 min. All interviews were audio‐recorded and transcribed verbatim. Relevant demographic data on the study participants (age, gender, race/ethnicity, marital status, living arrangements and education level) were collected from the Pepper Registry database.

### Data analysis

The accuracy of transcripts was verified with the audio recordings by two research team members. Transcripts were analysed using a thematic content analysis approach. This approach involved extensive content analyses to categorize the data into codes and then identify prevalent codes and themes.^21^ The interview questions were used as an initial guide to identify potential codes within the data. As coding continued, more subcategories and other prevalent codes emerged from the data. Coding was conducted using NVivo analysis software (version 11; QSR International, Doncaster, Victoria, Australia). Two members of the research team coded the transcripts. The research team met biweekly to refine and discuss frequently occurring codes and themes, and address any inconsistencies in the data. The final set of codes were then categorized and further merged into four central themes and assessed relative to features of sleep aid use. Given the small sample and qualitative findings, analyses were primarily descriptive.

## Results

### Participant characteristics

Of the 148 survey respondents eligible for this follow‐up study, 116 completed interviews with a response rate of 78.4%. Participant demographic characteristics are shown in Table 1. Nearly half of participants were aged 70–79 (47%), 29% were aged 80–89, and 9% were aged 90 or older. Almost two‐thirds of participants were women (65%), and the majority were white (95%). Over half of the participants were married (63%) and 69% were living with someone. Nearly half of participants had obtained a graduate or professional degree (47%), while 13% had only a high school diploma/GED or completed some high school education.

**Table 1:** Demographics of study participants

Characteristic	*N* (%)
Age	
60–69	17 (15)
70–79	55 (47)
80–89	34 (29)
90+	10 (9)
Gender	
Female	75 (65)
Male	41 (35)
Race	
Non‐Hispanic White	110 (95)
Non‐Hispanic Black	4 (3)
American Indian and Alaskan Native	1 (1)
Other	1 (1)
Marital status^a^	
Married	73 (63)
Divorced	12 (10)
Single	7 (6)
Widowed/widower	23 (20)
Living arrangements	
Living alone	36 (31)
Living with someone	80 (69)
Education^a^	
Some high school education	14 (12)
High school diploma/GED	1 (1)
Vocational school	3 (3)
College degree	41 (35)
Graduate/professional degree	55 (47)

a Missing data for marital status (one participant) and education status (two participants).

### Common Non‐prescription Sleep Aids

Participants reported the use of specific non‐prescription sleep aids including antihistamine‐only (31%), melatonin (30%) and combination antihistamine/analgesics (29%). Among the 36 antihistamine‐only products being used, 27 contained only diphenhydramine and nine contained only doxylamine. Among the 34 antihistamine–analgesic combination products reported, 22 were acetaminophen/diphenhydramine, 10 were ibuprofen/diphenhydramine, one was naproxen/diphenhydramine, and one was acetaminophen/chlorpheniramine.

Four major themes emerged including (1) experiences with sleep problems, (2) selection of non‐prescription sleep aids, (3) non‐prescription sleep aid use and (4) interactions with healthcare professionals. Themes and related verbatim quotes from participants are presented in Table 2.

**Table 2:** Prevalent themes, subthemes and verbatim quotes

Theme 1. Experiences with Sleep Problems	
Subtheme 1a. Causation	‘Sometimes when I'm thinking about things, about what's going on the next day, things revolve around and around in my mind and so that— And I find as I think more and more trying to solve the issues or solve what I'm going to be doing or a problem, I get so that I can't fall asleep’.
	‘Some nights I have a hard time going to sleep, and other times, well, as I've gotten older, I have to get up a couple of times during the night to go to bathroom. So that's a problem, because sometimes I can't go back to sleep after I go to the bathroom’.
	‘Another cause would be that I suffer from back pain. And so just the pain itself will wake me up or keep me from getting comfortable so that I can get to sleep’.
Subtheme 1b. Lifetime Experience	‘I have had them for probably thirty years. But they seem to get worse as I get older’.
	‘It's probably grown since about 10 years ago, gradually, and it's really annoying now. I think I'm being sleep deprived’.
Subtheme 1c. Impact on Quality of Life	‘I don't think it puts me in a bad mood or anything. I'm generally in a pretty good mood. I just think it… you know when you feel drowsy, you're just not as, you know, you're just not as productive, you're not as interactive’.
	‘So it doesn't seem to affect my daily schedule that much, or if it does I keep going regardless. So I don't know, it's a total puzzle to me it's a puzzle to my doctor’.
	‘I haven't really talked to anybody about problems with sleeping, because that doesn't seem to be a big issue at this point. It comes and goes’.
Subtheme 1d. Non‐pharmacological Approaches to Improve Sleep	‘Well, when I can't fall asleep, I do read a book’.
Theme 2. Selection of Non‐prescription Sleep Aids	
Subtheme 2a. Self‐recommendation	‘Well, since I was having these issues, I mean I frankly did not talk to a physician about this, I just decided to try something like that, and see if it helped, and it did. So it was more by, you know, trial and error. It wasn't like a systematic, somebody told me to do it’.
	‘Just on my own, just decided to try it and see if it helps and it did help, there's no doubt about that. So I just kept using it’.
Subtheme 2b. No Recognized Thought Process	‘You know as you walk in to the drug store and you look for sleep aids and I just saw something that said, “Help sleep,” and I purchased it’.
Subtheme 2c. No Consultation with Healthcare Provider	‘I told him I was taking it. You know, they ask for your medicines, and I put it on my list of medicines, and so I told—I didn't ask—he didn't suggest it in other words. I was already doing it myself’.
Subtheme 2d. Read the Label During Selection	‘When I buy it, yes, I read everything on the label’.
Subtheme 2e. Branding/Media	‘I guess I probably saw an ad or something. There was never a doctor or pharmacist that recommend I should take it’.
	‘Because I specifically went looking for it at a store that I don't even shop at because of the ad I saw on the internet’.
Theme 3. Non‐prescription Sleep Aid Use	
Subtheme 3a. Frequent Use	‘I take it almost every night, and the only reason I don't take it sometimes is because I'm out of it and didn't get to the drug store’.
	‘Yeah, I use it every night before I go to bed, just one pill’.
Subtheme 3b. Use for Longer than One Year	‘The first time I probably used it might have been even 20 years ago. At first maybe I just used it on occasion, and then gradually I've started using it more and more, so I can't really say—now, it's almost an every night thing. But I can't really say. Probably for the last four or five years, I might have used it every night’.
	‘Oh I would say I've been using that for, oh, I don't know how many years now. Been quite a while’.
Subtheme 3c. Read the Label Before Use	‘I don't know. I didn't read the instructions I just—there's two capsules in one little thing, one packet—and I just take it about a half hour before sleep with water. And I unfortunately didn't read what you're supposed to do’.
	‘I looked for directions to take, time, length to take, side effects, adverse reactions, all of that. That I look for, for every medication’.
	‘Well again, if I could read it, if it were printed large enough, I would have less trouble’.
Subtheme 3d. Not Taking as Directed	‘I never really paid any attention to the directions. I take a couple before I go to bed, about twenty minutes before I go to sleep, I go upstairs and go to bed. That's it’.
	‘I've been in the habit of taking some wine with supper, so I don't take it with the pill, but I take it a couple, three, four hours later’.
Subtheme 3e. Satisfaction with Use	‘Well, pretty satisfied. It does help me fall asleep and stay asleep, and go back to sleep when I invariably get up once or twice a night’.
	‘Well I think if I didn't take it I wouldn't sleep very well. So I'm satisfied’.
Subtheme 3f. Quality of Sleep with Use	‘Oh absolutely, absolutely, there is a dramatic difference when I use it versus when I don't’.
	‘Well I think I fall asleep faster and I don't wake up as much during the night’.
	‘It's pretty hard to say because I don't know how it would be if I didn't take it. But it definitely—it doesn't seem to make a lot of difference, but I figure it doesn't hurt me on the other hand’.
Subtheme 3g. Side Effects	‘It doesn't have much side effect. I mean, if you read the literature. So, no. I haven't complained about side effects’.
	‘Like I said, in the morning I do still feel the effects from it. Other than that, I don't think I feel any other side effects from it’.
	‘I think sometimes if I've taken it, it's harder to wake up in the morning’.
Theme 4. Interactions with Healthcare Professionals	
Subtheme 4a. Discussion About Sleep Problems	‘As a matter of fact I have. I've talked it over with my primary care physician’.
	‘I did mention it to my primary care physician. I definitely mentioned it after the breast cancer to my medical oncologist. He's the one that told me about melatonin’.
Subtheme 4b. Discussion About Non‐ prescription Sleep Aid Use	‘I go every six months to see him [physician] and I don't recall ever telling him that I took something as a sleep aid. And I don't think he ever asked’.
	‘Well no discussion, I haven't really had any discussions with anybody. I mean I know myself from my own knowledge, what the side effects are and what they can do and so forth. So I haven't had any discussions with a doctor or anybody’.
	‘No one discussed side‐effects with me. I knew about the side‐effects from a paper that I had read once written by a doctor concerning the dangers of acetaminophen’.
Subtheme 4c. Perceptions of Community Pharmacists	‘I would prefer to talk to my doctor because, as I said, he's been my doctor for several years and he knows my conditions and everything about me’.
	‘He's a pharmacist and he mainly fills prescription, and I think he would know a lot about prescriptions, as to what medication would go with another medication, but I don't really think he knows enough about—I don't think any pharmacist knows enough about the person's health, to make such judgments’.
	‘If I had questions, I would have no trouble at all in talking to him [the pharmacist]. Because I know they're quite knowledgeable about medications and the proper way to take them and the dosages and side effects. So I would have no problem talking to them if I did have questions’.

#### Experiences with sleep problems

Participants stated a variety of causes they considered to be associated with their sleep problems. Participant‐reported causes of sleep problems included worrying and stress (28%), caffeine use (22%), frequent night‐time bathroom use (20%) and other non‐sleep‐related ailments, like comorbidities (16%) and pain (16%). However, older adults most commonly reported being unsure of the cause of their sleep problems (29%).

Approximately one in five participants stated that their sleep problems had lasted over 10 years. Most participants reported that their sleep problems did not affect their mood (65%) or daily life (54%), but some participants said that their mood was negatively impacted or they were chronically tired from their sleep problems (28% and 23%, respectively). A majority of participants assumed their sleep problem did not require medical attention. Although participants used non‐prescription sleep aids, 23% also used reading as a non‐pharmacological approach to improve sleep.

#### Selection of non‐prescription sleep aids

More than twice as many participants chose their sleep aid based on self‐recommendation rather than by consulting with a healthcare professional (36% versus 16%). Just over one in five participants compared different non‐prescription sleep aids during the decision‐making process (22%). Many participants did not mention a specific rationale for selection of a particular sleep aid, and very few participants had consulted with a healthcare professional about selection. Exactly half of the participants reported reading the sleep aid label at least once before making a selection. Familiarity through branding or media was also a factor in some participants’ choice of non‐prescription sleep aids.

#### Non‐prescription sleep aid use

Forty percent of participants reported daily or frequent use of a non‐prescription sleep aid, and 56% reported using a non‐prescription sleep aid for more than one year. Few participants reported reading the non‐prescription sleep aid label regarding dosage information (22%) or side effects and warnings (19%), and many reported they had ignored or disregarded directions. Some participants mentioned the need for the labels to have larger print (17%). Nearly half of participants reported consuming alcohol on the same nights they used their sleep aids (45%). Participants were typically satisfied with their non‐prescription sleep aid (54%) and perceived improved quality of sleep with use (53%). Some participants reported being neither satisfied nor dissatisfied with their non‐prescription sleep aid (20%) and were unsure of the resulting quality of sleep (16%). Most participants reported they did not experience any side effects after taking the non‐prescription sleep aid; however, those that stated they did typically had drowsiness the following morning and experienced difficulty in waking up.

#### Interactions with healthcare professionals

While the majority of participants reported they had discussed their sleep problems with a healthcare professional (66%), far fewer had received consultation about non‐prescription sleep aid use (22%). Nearly one‐third of participants reported that their healthcare professionals were not aware of their non‐prescription sleep aid use (29%), and almost half (47%) had not discussed the safe use and side effects of non‐prescription sleep aids with their physician or pharmacist. The majority of participants reported they did not consult a pharmacist about their sleep problems (65%). One‐third of participants reported that they preferred provision of counselling related to sleep health from a physician rather than a pharmacist. A little over 20% of participants perceived that pharmacists only address medication concerns and not other aspects of health. About 24% of participants reported being comfortable with pharmacists, and 16% perceived pharmacists as being educated. Most participants felt that privacy was not a concern when discussing sleep problems and treatment with a community pharmacist.

## Discussion

Findings indicate that older adults encounter sleep problems due to various causes, and may often be self‐treating with non‐prescription sleep aids without awareness of safety risks or consultation with a healthcare professional.

### Strengths and limitations

The strengths of this study include the use of semistructured interviews that allowed for further discussion and clarification of questions with participants. Study findings contribute valuable evidence to the literature regarding older adults’ perspectives about their use of non‐prescription sleep aids. This study also has recognized limitations. Results may not be generalizable, because the study sample was drawn from a research registry of older adults living only in the Pittsburgh, Pennsylvania region, in the United States. Participants were predominantly female, White and well educated and thus are not representative of all older adults. Our qualitative study did not allow for determination of associations between participant characteristics and satisfaction with sleep aid use. We did not examine patient history or concurrent medication use, which may affect older adults’ use of non‐prescription sleep aids. Additionally, the usefulness of the information given by healthcare professionals was not evaluated.

### Sleep problems

Participants reported causes of difficulty falling or staying asleep which included anxiety, caffeine intake, bathroom use, comorbidities and pain, although some participants did not know the cause of their sleep problems. Older adults are typically more medically complex,^22^ and other underlying conditions may be contributing to their sleep problems. Past research has shown that chronic insomnia is likely a result of underlying health conditions, such as anxiety, depression and pain.^23^ Nearly one‐third of study participants reported taking an antihistamine/analgesic combination as a sleep aid, which may indicate that some older adults may be treating pain along with their sleep problems.

### Influence of pain and comorbidities on sleep problems

Participants reported the use of pain relievers/fever reducers without diphenhydramine to improve their sleep. The use of such products may suggest that pain is negatively impacting sleep in older adults, and they are self‐treating their pain using OTC medications. This was further supported by our study findings, as 16% of participants reported pain and 16% reported non‐sleep‐related ailments such as comorbidities to be causes of their sleep problems. Determining the possible contribution of comorbidities and pain to diminished sleep quality may likely be important in addressing older adults’ sleep problems and identifying appropriate treatments.

### Decision‐making process in selecting non‐prescription sleep aids

Our study showed that more participants were selecting non‐prescription sleep aids based on self‐recommendation rather than by consulting a healthcare professional, although community pharmacists might be present at purchase. Some participants noted being familiar with brands of certain non‐prescription sleep aids through advertising or media which significantly influenced their decision‐making process and selection. Knowledge of a non‐prescription sleep aid may be based on advertisements that could be misleading^24^ or portray non‐prescriptions as simple solutions to problems.^25^ Additionally, patients may assume there are fewer risks to taking a non‐prescription product if it can be obtained without a prescription.^26^ Perhaps partly due to this assumption, participants in our study typically did not compare non‐prescription sleep aids during selection, and only half reported reading the label. Further attention to details on dosage administration instructions, side effects and patient safety risks could help to inform older adults to avoid potentially inappropriate non‐prescription sleep aids.

### Non‐prescription sleep aid use

Study participants were typically satisfied with their sleep aids and perceived improved quality of sleep with use. Participants primarily reported taking non‐prescription sleep aids containing diphenhydramine and doxylamine, which the Beers Criteria for Potentially Inappropriate Medication Use in Older Adults strongly recommends avoidance of due to possible harmful effects.^15^ Nearly one‐third of participants reported they had taken melatonin, which is considered a dietary supplement. Melatonin is considered to be safe only for short term use 27 and is not regulated by the Food & Drug Administration (FDA). There is limited research on the long‐term effects of using melatonin to treat sleep problems; therefore, the safety of use on a chronic basis is not well known.

### Unsafe non‐prescription sleep aid use

Many unsafe behaviours related to non‐prescription sleep aid use were reported. Although non‐prescription sleep aids are not meant to treat chronic sleep problems, nearly half of participants used them daily or very often, and more than half reported use for more than a year. Few participants reported consulting the label for dosage information, and even fewer for side effects and warnings. This could possibly result in older adults taking non‐prescription sleep aids at a higher than recommended dose, for a longer duration, and without awareness of potentially dangerous side effects or interactions.

Nearly 40% of older adults in the United States are taking five or more prescription medications.^28^ Diphenhydramine has been found to have potentially harmful drug–drug interactions with prescription medications, including drugs used to treat breast cancer (tamoxifen),^29^ hypertension (metoprolol)^30^ and depression (venlafaxine).^31^ Furthermore, concurrent use of prescription sleep aids, such as zolpidem and temazepam, and OTC sleep medications containing diphenhydramine or doxylamine could lead to increased risk of central nervous system depression. Also, melatonin has been found to reduce the effectiveness of calcium channel blockers such as amlodipine.^32^

Although alcohol can exacerbate the sedative effects of non‐prescription sleep aids, nearly half of participants reported consuming alcohol on the same nights as sleep aids. Many sleep medications have been contraindicated with alcohol use due to unsafe interactions.^33^ Alcohol may dangerously intensify the effects of ingredients in common sleep medications.^33^ This is an important patient safety concern because many participants reported they did not read the medication information labels and neglected to follow their directions. Furthermore, study participants’ perceptions of sleep problems may be influenced by the concurrent consumption of alcohol and a non‐prescription sleep aid.

Medication labels on non‐prescription sleep aids containing diphenhydramine or doxylamine warn that patients should consult a doctor if they need to use the sleep aid for longer than two weeks and alcohol should not be consumed with use. However, the text size is often small and some study participants reported experiencing trouble reading such information. Older adults with lower levels of health literacy may also have more difficulty understanding medication label information. Larger and clearer font 34 and simpler language 35 could possibly lead to better understanding of non‐prescription sleep aid instructions and potential risks.

### Consultations with pharmacists

Older adults could receive consultation about their sleep hygiene and safe non‐prescription medication use from healthcare professionals such as doctors, nurses or pharmacists. Community pharmacists are medication experts that are easily accessible where non‐prescription sleep aids are sold; however, few of the participants in this study had used them as a resource for discussing their sleep problems and receiving information about appropriate OTC medication use. This may be due in part to the belief that community pharmacists primarily address questions about prescription medications and not health concerns. Often, participants reported they typically would discuss sleep problems with their physicians and did not think of asking pharmacists about health or medication concerns. However, some participants stated that they would be comfortable discussing health concerns with pharmacists and considered them to be well educated. Overcoming these perceptions of community pharmacists could potentially encourage older adults to seek medication counselling from these healthcare professionals when selecting and purchasing non‐prescription sleep aids in pharmacies to improve patient safety.^36^

## Conclusions

Findings indicated that many older adults experience sleep problems due to various causes, often from worrying, stress or caffeine use. Many older adults self‐treat sleep problems with non‐prescription sleep aids and are often taking potentially inappropriate products containing diphenhydramine or doxylamine, and many are doing so for longer than these products are intended to be used. Over half of participants reported using a non‐prescription sleep aid for more than one year, were satisfied with its use and perceived it improved sleep quality. The decision‐making processes in choosing non‐prescription sleep aids do not appear to involve appropriate attention to dangerous risks and side effects on the medication label, with frequent disregard to directions and minimal healthcare professional consultation. Community pharmacists are easily accessible and able to provide medication counselling related to non‐prescription sleep aid use, but patients tend to perceive them as medication dispensers, rather than resources for addressing health or medication‐related concerns. Future investigations should focus on development of patient‐centred interventions to facilitate patient–pharmacist engagement in community pharmacies to support older adults’ safe and appropriate treatment of sleep problems, while minimizing the risks of potentially inappropriate non‐prescription sleep aids containing diphenhydramine or doxylamine.

## Declarations

### Conflict of interest

The Author(s) declare(s) that they have no conflicts of interest to disclose.

### Funding

This work was supported by the Academy Health New Investigator Small Grant Program (NISGP).

### Authors’ contributions

All authors contributed to (1) study design; (2) collection, analysis and interpretation of data; (3) the writing of this report; and (4) the decision to submit the manuscript for publication. All Authors state that they had complete access to the study data that support the publication.

## Supporting information


**Appendix S1.** Older Adult Interview Guide.
