# Supplementary material for: Managing sleep problems using non‐prescription medications and the role of community pharmacists: older adults’ perspectives
Source: Int J Pharm Pract. 2017 Mar 6;25(6):438–46. doi: 10.1111/ijpp.12334 (PMC5724494; doi:10.1111/ijpp.12334)
Supplement: Supplementary file 1 — Appendix S1. Older Adult Interview Guide. [file IJPP-25-438-s001.docx]

**Supplementary Material**

**OLDER ADULT INTERVIEW GUIDE**

**Hello this is _________________. I’m calling from the University of Pittsburgh on behalf of The Claude D. Pepper Center.**

*You recently completed a questionnaire from the Claude D. Pepper Center and we would like to follow-up with a few questions about managing your sleep health. As part of this interview we will be asking details about any over-the-counter or OTC sleep aids that you may be using to help you fall asleep or stay asleep. We would appreciate it if you could have these OTC sleep aids with you during the interview. Thank you! We will now begin with a few questions about your sleep in general.*

1. Would you say that you ever have trouble sleeping, either falling asleep or staying asleep within the past year?

2. Can you describe your sleep problems and how long you have had them?

3. What do you feel causes your sleep problems?

4. How have your sleep problems affected your day-to-day activities, such as your work, seeing friends and family, or hobbies?

5. How have your sleep problems affected your day-to-day mood?

**Now I want to ask you some questions about things you may have done about your sleep problems.**

6. Have you ever discussed your sleep problems with a healthcare professional, like a doctor, pharmacist, or nurse practitioner?

7. Was this healthcare professional your community pharmacist?

8. Did they provide any non-drug and/or drug treatment or advice for your sleep problems?

9. Do you think there are any ways the community pharmacist could help you deal with your sleep issues?

10. Are there any reasons you don’t discuss your sleep problems with your community pharmacist?

11. Do you feel that your community pharmacist is an appropriate person to talk to about using prescription or OTC medications?

12. Do you feel that your community pharmacist is an appropriate person to talk to about your health conditions and concerns?

13. How comfortable would you be approaching your community pharmacist with a question about your health conditions or concerns?

14. Would you be more comfortable if you had a private area to discuss your health concerns with your community pharmacist?

**The next few questions I’m going to ask about your use of OTC sleep aids.**

15. What OTC sleep aids do you currently use?

16. How long have you been using this sleep aid?

17. How did you decide to start using this OTC sleep aid?

18. How satisfied are you with your experiences taking this sleep aid?

19. Do you feel that this sleep aid has improved your sleep quality?

20. How has taking this sleep aid improved your sleep quality?

21. Do you ever feel side effects from taking this sleep aid?

22. Do you feel drowsy in the morning after taking any of this OTC sleep aid?

23. When choosing this sleep aid, did you consult the label?

24. What information did you look for on the label?

25. How would you improve the label so it’s easy to understand the directions?

26. Do you ever drink alcohol on the same nights as taking an OTC sleep aid?

27. Did you compare this sleep aid with other available OTC sleep aids when choosing this one?

28. Why did you choose this particular OTC sleep aid over other OTC sleep aids?

29. Did you talk to a doctor, pharmacist, or other medical professional about this sleep aid before you started taking it?

30. Did they discuss safe use or side effects of your OTC sleep aid?

31. Does your doctor know you are currently taking this sleep aid?

**Many people use methods other than prescription medicines or OTC sleep aids to help improve their sleep quality. We would now like to ask you about some of the other things you might do to help you fall asleep, stay asleep, or otherwise sleep better.**

32. Do you use any methods other than prescription medicines or OTC sleep aids to help improve your sleep quality?

33. What are some of the methods you use to help improve your sleep quality?
